# Gene Network Biological Validity Based on Gene-Gene Interaction Relevance

**DOI:** 10.1155/2014/540679

**Published:** 2014-09-08

**Authors:** Francisco Gómez-Vela, Norberto Díaz-Díaz

**Affiliations:** School of Engineering, Pablo de Olavide University, 41013 Seville, Spain

## Abstract

In recent years, gene networks have become one of the most useful tools for modeling biological processes. Many inference gene network algorithms have been developed as techniques for extracting knowledge from gene expression data. Ensuring the reliability of the inferred gene relationships is a crucial task in any study in order to prove that the algorithms used are precise. Usually, this validation process can be carried out using prior biological knowledge. The metabolic pathways stored in KEGG are one of the most widely used knowledgeable sources for analyzing relationships between genes. This paper introduces a new methodology, GeneNetVal, to assess the biological validity of gene networks based on the relevance of the gene-gene interactions stored in KEGG metabolic pathways. Hence, a complete KEGG pathway conversion into a gene association network and a new matching distance based on gene-gene interaction relevance are proposed. The performance of GeneNetVal was established with three different experiments. Firstly, our proposal is tested in a comparative ROC analysis. Secondly, a randomness study is presented to show the behavior of GeneNetVal when the noise is increased in the input network. Finally, the ability of GeneNetVal to detect biological functionality of the network is shown.

## 1. Background

Modeling process occurring in living organisms is one of the main goals in bioinformatics [1–4]. Gene networks (GNs) have become one of the most important approaches to discover which gene-gene relationships are involved in a specific biological process.

A GN can be represented as a graph where genes, proteins, and/or metabolites are represented as nodes and their relationships as edges [1].

It is important to note that GNs can vary substantially depending on the model architecture used to infer the network. These models can be categorized into four main approaches according to Hecker et al. [1]: correlation [5, 6], logical [7–9], differential equation-based, and Bayesian networks [10, 11]. These approaches have been broadly used in bioinformatics. For example, Rangel et al. [12] used linear modeling to infer T-cell activation from temporal gene expression data, or Faith et al. [13] adapted correlation and Bayesian networks to develop a method for inferring the regulatory interactions of* Escherichia coli*.

Once a model has been generated, it is very important to assure the algorithm reliability in order to demonstrate its efficacy. The quality of the algorithm(s) can be measured by applying so-called synthetic data [14] and/or by using prior biological knowledge [15]. Synthetic data approaches can be used to analyze the performance of the GN inference algorithm, whereas a study of biological validity is supported by real data.

Synthetic data methods produce an artificial data set according to a previously known network. The values of the simulated gene expression are stored in a data set and used as input for the GN inference algorithm. Finally, the performance of the algorithm is tested comparing both GNs. Currently, this process can be carried out using different tools as GeneNetWeaver [16] or SynTReN [17].

Although this approach is commonly used for comparing inference algorithms, it can not fully reproduce the internal features of real biological processes. This drawback means they are not suitable for the validation of the inferred models, from a biological point of view.

To address this issue, comparison with prior biological knowledge has been proposed [18, 19]. Currently, there are a number of different available biological repositories where the Kyoto encyclopedia of genes and genomes (KEGG) is one of the most widely used for analyzing relationships between genes [20, 21]. KEGG's metabolic pathways contain knowledge about different biological processes. These pathways are represented as a graph where nodes represent genes, enzymes, or compounds (i.e., carbohydrates, lipids, and amino acids) and edges encode relationships, reactions, or interactions between the nodes. The pathways contained in the KEGG database represent the actual knowledge of molecular interaction and reaction networks for metabolism, genetic information processing, environmental information processing, cellular processes, and human disease. They provide useful structured information for gene network validation. For example, C. Li and H. Li [15] used KEGG transcriptional pathways to perform a network analysis of the glioblastoma microarray data, or Ko et al. [22] tested a new Bayesian network approach using gene-gene relationships stored in KEGG. In this line we proposed a GN validation framework based on a direct comparison between a gene network and KEGG pathways [23].

The aforementioned approaches, hereafter called the classical use of KEGG, present three major shortcomings: (a) not all the biological information is used, (b) only strong gene-gene relationships are considered, and (c) the current biological knowledge is not complete.

Gene-gene relationships are only usually considered by metabolic pathways based GN validation approaches. Hence, all other biological information provided by pathways is ignored, such as gene-compound or compound-compound relationships (see Table 1). For example, Wei and Li [24] only used human gene-gene interactions stored in the KEGG pathways when performing simulation studies, excluding gene-compound and compound-compound relationships. Or Zhou and Wong [25] used the relationship between KEGG gene pairs (mainly PPrel and ECrel) to study protein-protein interaction data sets.

Furthermore, current GN validation approaches are not entirely accurate as they only consider strong relationships between genes (direct gene-gene interactions), leaving weaker relationships to one side [4].

In addition, the use of prior biological knowledge could present another important lack, the current limitations of the biological databases. As described by Dougherty and Shmulevich [2], biological knowledge has some intrinsic limitations in the sense that they depend inherently on the nature of scientific knowledge. Others are contingent depending on the present state of knowledge, including technology. Current validation methods use these biological databases in order to classify the inferred relationships as true or false positives. Due to the intrinsic problem of the biological databases, it is not possible to argue that these false positives are actually caused by a bad prediction from the inference methods or because of incomplete knowledge.

This paper proposes a new methodology, GeneNetVal, to analyze the biological validity of a gene network by utilizing the biological information stored in KEGG by weighting the gene-gene relationships. GeneNetVal uses different types of relationships contained in KEGG pathways (gene-gene, gene-compound, and compound-compound), carrying out an exhaustive and complete conversion of a pathway into a gene network. The network obtained will be used as a gold standard in comparison with the input network. Moreover, a novel matching distance is proposed. This measure, based on gene-gene interaction relevance, takes into account the concept of weak relationships between a pair of genes to present a set of nondeterministic indices with different levels of accuracy. Thus, we do not categorically accept or refuse a gene-gene relationship, but a weighted value is assigned according to distance of those genes in the pathway. Through these values we generated a new gene network validity measure and mitigate the problem of the incomplete biological knowledge.

## 2. Methods

In this section, the GeneNetVal methodology and also the methods used to perform the experiments will be presented. These methods will be used in Results and Discussion section.

### 2.1. GeneNetVal Methodology

**Figure 1:**
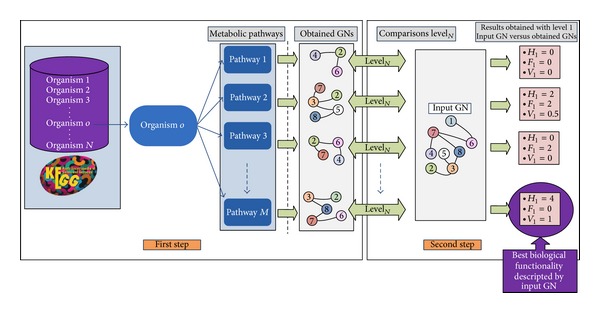
A schematic representation of GeneNetVal methodology. In the first step, organism o's information is extracted from KEGG database. Each of the* M *metabolic pathways is processed to obtain* M *gene networks. In the second step,* M *evaluations of the input network are carried out. Note that the results presented were obtained by applying our approach at level 1.

As already stated, the two-step methodology proposed, GeneNetVal, is based on KEGG metabolic pathways and summarized in Figure 1. In the first step, a complete conversion of a metabolic pathway into a gene association network is carried out. In the second step, the biological validity of a GN is determined. In order to do this, a novel matching distance between networks is used.

#### 2.1.1. Step One: From Metabolic Pathways to Gene Association Networks

KEGG database stores knowledge about many different organisms, but we only need the information pertaining to the network to be analyzed. Hence, only the KEGG metabolic pathways for the same organism of the input network are considered. This is represented in Figure 1, where all pathways of the organism *o* are extracted.

These pathways are converted into gene association networks where all types of pathway relationships (see Table 1), including gene-gene (PPrel, ECrel, and GErel), gene-compound (PCrel), and compound-compound, are used.

**Figure 2:**
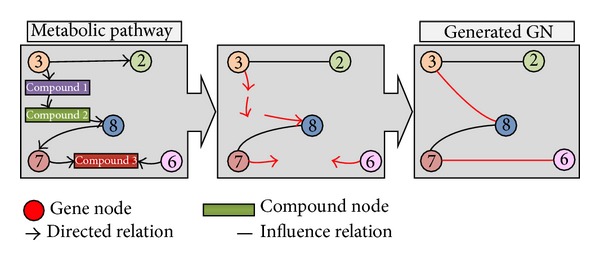
The simplest conversion example. In the first substep the compound nodes and the direction of the relationship edges are removed. In the second substep, new association relationships are established.

As stated previously, a metabolic pathway is composed of different types of nodes (genes or other compounds) while genes are only used in gene networks. This difference exhibits that direct comparison between them is unreliable based on the information containing different elements. This difference is overcome by increasing the abstraction level of the pathways. Concretely, each pathway is converted into a gene association network, the highest level of abstraction for reconstruction of gene regulatory processes as it is described by Martínez-Ballesteros et al. [30]. This conversion process is represented in Figure 2 and explained bellow.

Firstly, all the compound nodes presented in the pathway are removed. However, gene nodes are conserved along with their relationships of influence (nondirect edges), be they PPrel, ECrel, or GErel. The PCrel, compound-compound, and others relationships are processed in different way.

The compound nodes located between two genes carry information from one gene to another. They act as a bridge between the genes, so these two gene nodes should be related. Based on this, after removing the compound nodes, new undirected gene-gene relationships will be created. These relationships are established between each pair of genes that were previously associated with the same compound node.

Figure 2 shows the conversion process from* “Pathway M”* (Figure 1) to a gene network in detail. For example, genes 3 and 8 are associated with a compound node in the pathway but there is no direct relationship between them. However, the information pertaining to this indirect gene-gene influence should be taken into account so that a new influence relationship between genes is created. Similarly, a relationship is generated between genes 6 and 7.

**Figure 3:**
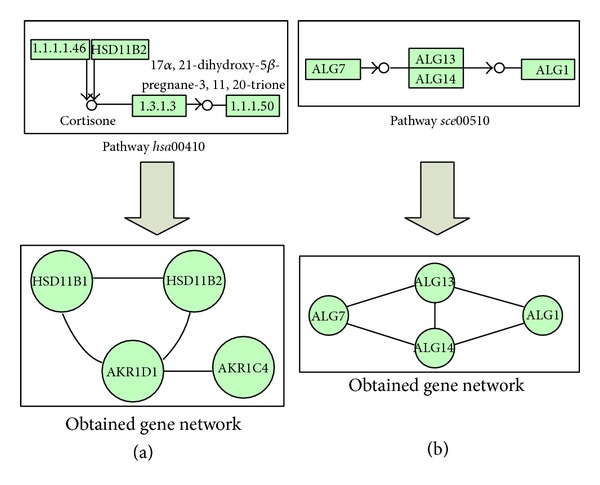
Two portions of real KEGG pathways with multiconnected nodes. (a) contains a fragment of the pathway *hsa*00410 where three genes are connected to the same compound. In the process of conversion to a gene network, new relationships between these genes are created. (b) shows a fragment of the *sce*00510 pathway, illustrating how a compound is transferred by two genes. When the gene network is created, these two genes must be connected (as shown in the figure). Note genes HSD11B1, AKR1D1, and AKR1C4 (a) correspond to enzymes “1.1.1.146,” “1.2.1.3,” and “1.1.1.50,” respectively.

The conversion presented in Figure 2 is a simple example; pathways are often more complex. In a pathway, multiple genes are likely related to the same compound node, or the chemical compounds are transferred by two or more genes/enzymes. These two cases should be considered to carry out an exhaustive conversion. In the first type, multiple genes somehow interact with the same compound (substrate of a chemical reaction, product, etc.). This biological information is preserved creating new relationships (see Figure 3(a)). In the second group, the genes responsible for transferring the compounds should be related in the new GN, since they actually interact with the chemical compounds simultaneously. Hence, new relationships between these genes are included (see Figure 3(b)).

#### 2.1.2. Second Step: Biological Validity

In the second step, the metabolic pathways are used as biological knowledge to evaluate the input network. Usually, the literature applies a scoring methodology [1, 27, 29] to evaluate an inferred model using prior knowledge, be it synthetic or biological data. Based on this idea and on the notion of the strong and weak relationships in GNs [4], the authors have developed a novel measure for evaluating the validity of an input network that is based on the relevance of the gene-gene interactions stored in KEGG.

Let *G*_*I*_ = {*N*_*I*_, *E*_*I*_} and *G*_*P*_ = {*N*_*P*_, *E*_*P*_} be two graphs, where (*N*_*I*_, *N*_*P*_) represent the nodes of the graphs and (*E*_*I*_, *E*_*P*_) represent the edges (gene-gene relationships). The validity of the input graph (*G*_*I*_), according to the biological information of pathway *P* represented in the graph *G*_*P*_, is measured as the difference between both graphs at certain level of distance.

**Definition 1 (Level).**

Let a graph *G* = {*N*, *E*} and two nodes *g*_*α*_, *g*_*β*_ ∈ *N*. The level of the relationship between (*g*_*α*_, *g*_*β*_) is calculated as the number of edges between nodes *g*_*α*_ and *g*_*β*_ in *G*.

**Figure 4:**
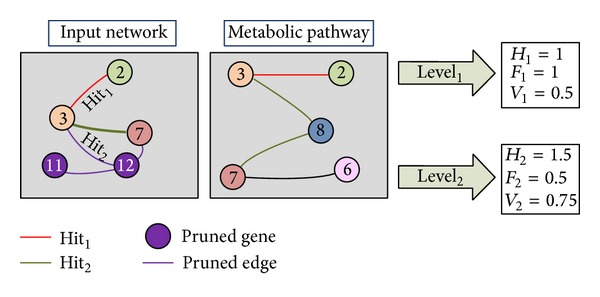
An example of the comparison using level 1 and level 2. Examples of Hit_1_ and Hit_2_ are presented. The purple nodes and their relationships are pruned for this specific evaluation because they do not belong to the metabolic pathway.

For example, in Figure 4, the relationship between nodes 3 and 7 in *G*_*P*_ has a level of 2 because there are two edges between these nodes.

**Definition 2 (*Hits at level l *(*Hit*_*l*_)).**

The number of edges where the level between the nodes directly connected in *G*_*I*_ is *l* in *G*_*P*_.

An example of Hit_1_ and Hit_2_ can be found in Figure 4, where the edge between genes 3 and 2 represents the Hit_1_ and the edge between 3 and 7 is Hit_2_. Obviously the greater the distance between nodes, the lower the relevance of the evaluated relationship. Thus, the new matching distance provides two weighted indices through comparison with the selected level.

**Definition 3 .**

Cumulative hits at level *n*, *H*_*n*_, can be defined as the weighted sum of correctly inferred edges at level *n* in *G*_*I*_ according to the information presented in *G*_*P*_. Consider

(1) $$\begin{array}{c} H_{n}\left(G_{I},G_{P}\right)=\overset{n}{\underset{l=1}{\sum }}\frac{\text{Hi}{\text{t}}_{l}}{l}, \end{array}$$

where *H*_*n*_ denotes the sum of edges that were correctly inferred weighted by their relevance in the network with distance (level) *n*.

Figure 4 presents an example of calculation of *H*_1_ and *H*_2_.

**Definition 4 .**

Cumulative failures at level *n*, *F*_*n*_, can be defined as the number of incorrect inferred edges at level *n* in *G*_*I*_

(2) $$\begin{array}{c} F_{n}\left(G_{I},G_{P}\right)=||E_{I}||-H_{n}\left(G_{I},G_{P}\right), \end{array}$$

where ||*E*_*I*_|| is the number of edges in *G*_*I*_. Thus, *F*_*n*_ denotes the number of edges that were not correctly inferred in the network with distance (level) *n*.

Figure 4 shows an example of calculation of *F*_1_ and *F*_2_. At level 1, the graph presents one cumulative failure because of the genes 3 and 7, which are directly connected in *G*_*I*_ and have a distance of 2 in *G*_*P*_. As the interaction between 3 and 7 is weak (hit of level 2), the value of the cumulative failure level 2 is 0.5. Accordingly, the validity measure can be defined.

**Definition 5 .**

The validity (GeneNetVal measure) of graph *G*_*I*_ according to *G*_*P*_ level *n*, *V*_*n*_, is defined as the proportion of correctly inferred edges at level *n* in *G*_*I*_. Consider

(3) $$\begin{array}{c} V_{n}\left(G_{I},G_{P}\right)=\frac{H_{n}\left(G_{I},G_{P}\right)}{H_{n}\left(G_{I},G_{P}\right)+F_{n}\left(G_{I},G_{P}\right)}=\frac{H_{n}\left(G_{I},G_{P}\right)}{||E_{I}||}. \end{array}$$

This measure ranges between 0 and 1, where 0 is the lowest validity value and 1 the highest. The validity measure estimates the ratio of correctness of *G*_*I*_ with respect to *G*_*P*_.

The biological validity is obtained as the proportion of positive prediction according to the cumulative hits and failures. This is the principal measure obtained by our methodology to rate the quality of a GN.

### 2.2. ROC Study

A receiver operating characteristic (ROC) analysis will be presented in the Results section. The goal of this study is to compare the performance of different gene network validity approaches, evaluating real networks against random networks (without biological sense). The three networks will be used in the experiment, attempt to encompass the regulation of a large number of functional processes in yeast. Hence, we have assumed that these networks contain biological meaning of each functional process described in the KEGG pathways (they are functionally complex networks).

Therefore, the evaluation of these networks should produce relevant validity results for each of the pathways considered. In contrast, the biological validity of random networks ought to yield poor results because, in fact, they should not contain biological meaning.

A validity threshold (*T*) has been used to decide if the input network has relevant information for each selected pathway.* T* denotes the minimum validity value for a network with a specific pathway to be considered as valid value. In order to generate the ROC curve for each experiment, we have used 101 different* T* values (from 0 to 1). A confusion matrix is obtained for each iteration. If the validity value obtained for a pathway exceeds the* T* value, the input network is classified as a positive (true positive or false positive, depending on whether the input network is a real network or a random network). If the obtained value is lower, the input network is described as a negative (true negative or false negative). With this idea, for each iteration the indexes are computed for the confusion matrix.

Hence, it is possible to calculate 101 confusion matrices and 101 true positive rates (TPR) and false positive rates (FPR) values to draw the curve.

**Figure 5:**
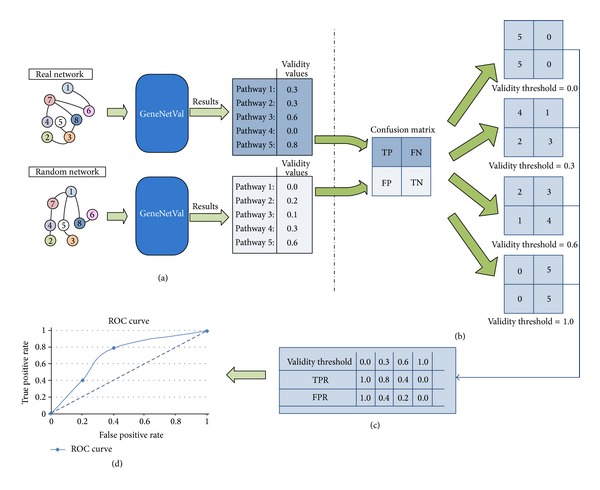
Representation of a toy example for the ROC study performed. (a) represents the GeneNetVal process, where the validity values for both networks are obtained. In (b) the confusion matrices are obtained. The TPR and FPR values are presented in (c). Finally the ROC curve is depicted in (d).

Figure 5 provides a toy example showing the entire process (only for one random network). It offers a comparison between the results obtained by a real network and the results obtained by a random network. With the validity values obtained for both networks (Figure 5(a)), different confusion matrices were generated according to different thresholds, only 4 thresholds in this example (Figure 5(b)). Thus, for each iteration it is possible to obtain the values of TPR and FPR (Figure 5(c)). With these values, the ROC curve is finally represented (Figure 5(d)).

**Figure 6:**
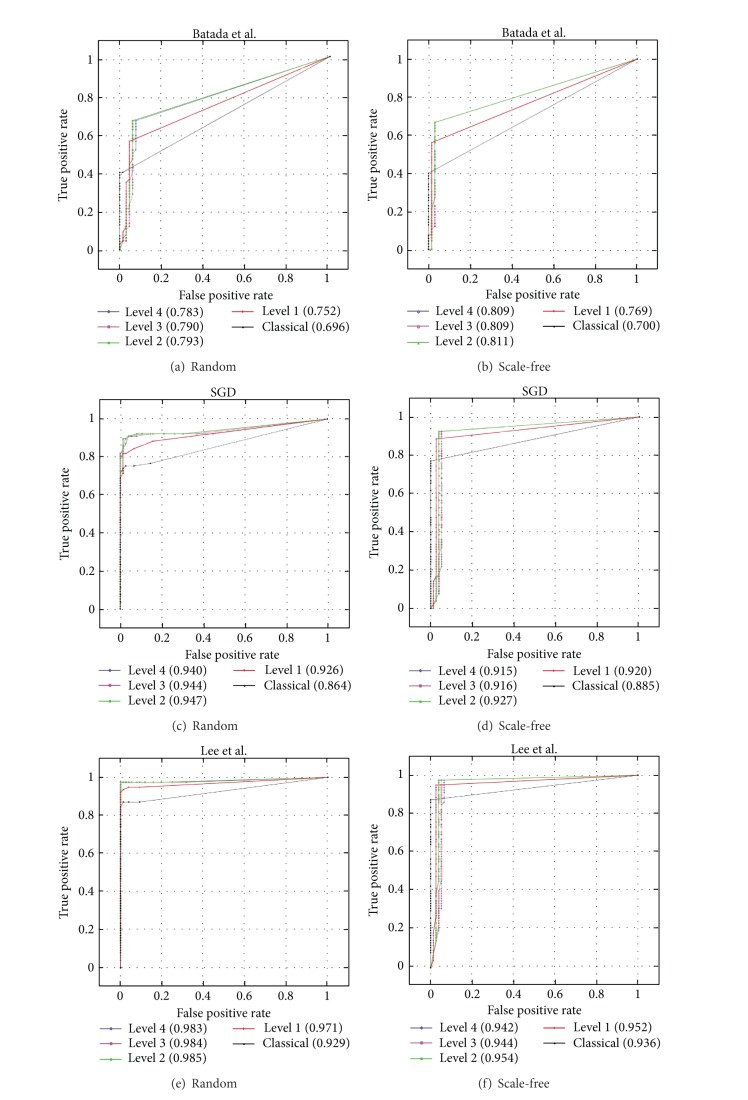
ROC analysis of our methodology using some yeast networks. For this analysis two different topologies were used: pure random and scale-free topology.

It is important to note that the results presented in Figure 6 are average values for a sample of random networks.

### 2.3. Selecting Functional Description with GeneNetVal

The specific functionality of the input network could be studied in accordance with the biological process information store in a specific KEGG pathway. A metabolic pathway represents a model of a particular biological process. Different sets of genes are involved in different pathways. This should be considered if a functional assessment of the input network is performed. If a pathway contains a set of genes, this set is annotated to the pathway's biological function. Hence, any information from the input network that does not belong to the specific biological process will be not taken into account for this validation. Note that these relationships should not be considered as a failure because actually there is no information to classify the validity of the interactions from genes in the input network that are not present in metabolic pathways.

This pruning process, which is depicted in Algorithm 1, entails removing any edge from the input network if the corresponding genes are not present in the specific pathway. The input network will suffer a different pruning for each pathway. Through this pruning, the input network can be evaluated independently for each process. An example of this pruning is shown in Figure 4 where the purple edges are removed for the comparison with the pathway.

After pruning, the comparisons with each pathway will show the validity measure. The functionality described by the pathway with the highest value of *V*_*n*_ (GeneNetVal measure) will be the functionality that best fits the input network. A high *V*_*n*_ value means that the input network fully or partially describes the functionality that is described by that particular metabolic pathway.

Hence, *M* different comparisons have been carried out in Figure 1, where the highest value was generated by the gene network extracted from “*pathw*a*yM*.”

It is also possible for the input network to contain information about more than one specific biological process. Alternatively, the biological processes are usually interrelated (e.g., the cell cycle and the meiosis). An example of this situation in Figure 1 might be the comparison between the gene network from “*pathw* *a* *y*2” and the input network. In that case, the highest values of the validity measure could be considered, to determine which processes are better described.

## 3. Results and Discussion

The performance of our proposal was tested through three experiments using different types of networks. Firstly our proposal was compared with the classical use of KEGG. A ROC analysis of different distance level of GeneNetVal and precision measure were carried out. The behavior of the method proposed with different noise level is tested in the second experiment. Finally, the ability of GeneNetVal to detect the biological functionality encoded in a input network is analyzed in the third experiment.

### 3.1. ROC Analysis

The ROC analysis was performed to show the improvement achieved by our approach over those that only consider direct gene-gene relationships [24, 25], along with its robustness against information without biological meaning (see Section 2.2).

ROC analysis has been widely used in the literature [31, 32] because it is able to score the performance of classifiers and rankers as a trade-off between a true positive rate and false positive rate. Additionally, the area under the ROC curve (AUC) is presented, as it provides information about the level of randomness of the approach.

For this study three complex and contrasting yeast gene networks with different types of gene relationships were used. A protein-protein interaction network was used by Batada et al. [33] in the analysis of highly connected proteins in a network (hubs). The network resulting of selecting the protein-protein and protein-DNA interactions of the Saccharomyces Genome Database (SGD) [34] provides an access to the complete* Saccharomyces cerevisiae* (yeast) genomic sequence. And, finally, the network was presented by Lee et al. [35] (YeastNet v.2) which combines protein-protein, protein-DNA, coexpression, phylogenetic conservation, and literature information.

For every input network explained above, two different topologies of random networks were considered: pure random and scale-free. This latter topology is used since biological networks usually follow it [36, 37].

The sample size for each input network and topology was calculated with a confidence interval of 95% for an infinite population of networks [38]. Hence, a sample size of 385 random networks was used. Pure random networks were designed to have the same node and edge size as the input network, but gene-gene relationships were randomly generated. Scale-free networks were generated using the open source library JGraphT, with the same nodes as well. To use information stored in KEGG, we extracted the KGML files of yeast pathways using the KEGG API.

The results of the analysis are represented in Figure 6, where each row represents the study of a different input network. The left column in the figure represents the study for pure random topology, and the rightmost shows the scale-free topology. Each graph contains five lines that encode the behavior of GeneNetVal considering the distance levels from one to four and the precision measure [30, 39] for the classical use of KEGG. In total, more than 11000 (3 input networks × 2 topologies × 5 measures/levels × 385 networks) evaluations were carried out.

The ROC curves show that the results of the three networks follow a similar pattern for both topologies. Particularly striking is the distance between the point (1, 1) and the one above. FPR is 1 for a threshold equal to zero (see Section 2.2 for more details) but represents a very low value for the next checkpoint (threshold = 0.01). This could be due to the fact that the use of KEGG as gold standard is very effective detecting interactions with no biological meaning.

For some levels the lines do not start at point (0, 0) (Figures 6(b) and 6(d)). This is because some KEGG pathways do not contain many interactions (e.g., *sce*00062 pathway contains only 5); so a random network might contain those gene relationships at a certain distance level.

Regarding the values obtained for the area under the curve (AUC), it is important to note that the major is the number of type of relationships considered in the network the better the methodology performs. The best results are obtained by Lee's network [35] which combines four different types of relationships. The second best result is generated using SGD, while Batada's network presents the worst result. This makes sense since KEGG pathways gather biological data from various contrast sources.

Comparing the classical use of KEGG with level 1 of our proposal, which only differs on how the pathways information is managed, is possible to argue that the conversion proposed produces significant improvement in AUC. Level 1 produces better result in all cases. For example, the AUC value of 0.88 is increased to 0.92 in SGD for scale-free topology (Figure 6(d)). Furthermore, it is possible to improve AUC by increasing the distance level in the comparison. The best result is shown by level 2, while levels 3 and 4 perform worse than levels 1 and 2.

The results presented show that GeneNetVal is capable of detecting gene relationships with and without biological meaning. Furthermore, the methodology presents a significant improvement compared to the classical approach (precision) for all levels studied. In particular, the best performance is obtained by level 2 for all the experiments.

Finally, in spite of the fact that biological databases are crucial information sources for evaluating results obtained in any study, they have some limitations. These limitations are intrinsic to all of them, in the sense that they depend inherently on the nature of scientific knowledge; others are contingent, depending on the present state of knowledge, including technology [2, 40]. Such limitations can include incorrect event or entity labels, missdirections in the relationships, absence of associations, and other ambiguities. Consequently, the performance of prior knowledge-based methods could be affected by these limitations, including our approach. In particular, GeneNetVal could be affected for incorrect event or entity labels and also for the absence of association in the metabolic pathways in terms of bad classification of relationships (incorrect hit or failure). Despite this fact, it is worth mentioning that the classical approaches are also affected for the problems presented above. In this sense, GeneNetVal presents a more robust performance than the classical approaches, since the use of indirect relationships mitigates these problems. This affirmation is supported by the results presented in this ROC analysis, where GeneNetVal performs better than the classical approach even though the same databases (containing the same lacks) are used in both methods.

### 3.2. Randomness Study

Despite the fact that in the ROC analysis section it was shown that GeneNetVal is better distinguishing real networks from random networks than a classical approach extracted from the literature, in this section the behavior of the methodology to the progressive inclusion of noise will be shown.

Concretely, we have carried out the study for all of the yeast networks which were previously presented in the paper (Batada, Lee, and SGD networks). These input networks were changed increasing randomness in their gene relationships. Hence, in a loop process composed of 10 iterations, the random relationships added to the networks were increased in 10% at each iteration. In the same way a 10% of the original relationships were removed. To avoid bias, this was done 385 times (sample size with a confidence interval of 95% assuming an infinite population of random networks) [38]. Therefore, 15360 (385 networks × 10 iterations × 4 original networks) different random networks were analyzed.

**Figure 7:**
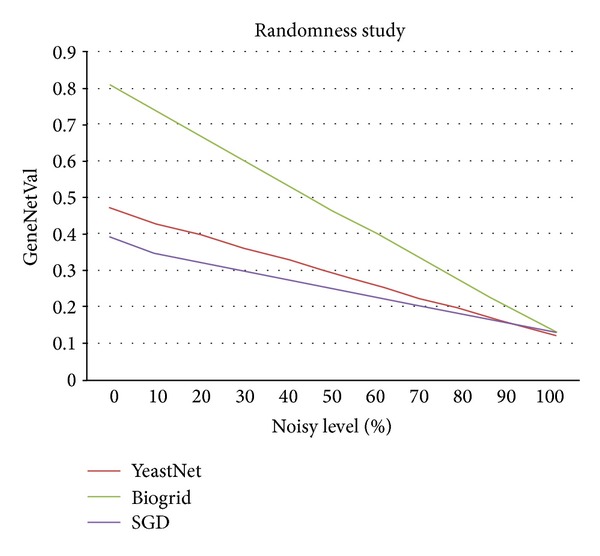
Results of the randomness study of GeneNetVal using level 2. For this study, we have used different yeast networks versus pathway* sce*04111.

According to the results presented in the ROC analysis section, the validity value level 2 was considered in this experiment. As gold standard, we have used the pathway *sce*04111 (yeast cell cycle) since it is one of the most studied pathways from yeast [41–43]. The results averages are summarized in Figure 7.

Figure 7 presents the evolution of the validity values for the yeast networks. It can be observed that the different validity values follow a similar behavior. This behavior verifies that the loss of relevant information in the networks is progressive, and it increases as the randomness is increased in them as well. These results show that our method is able to detect the loss of information as the randomness increases in the networks.

### 3.3. A Functional Study: Yeast Cell Cycle Networks

In this section some well-known yeast networks are used to prove the usefulness of our approach by detecting specific biological functionality as it was described in Section 2.3. These networks were produced by applying different gene networks inference approaches to the same time-series yeast cell-cycle microarray [44]. Concretely, the networks were generated by applying the approaches of the network presented by Nariai et al. [26], which is obtained through a Bayesian-based algorithm; Bulashevska and Eils [28] that is another Bayesian-based algorithm; Ponzoni et al. [29] whose algorithm called GRNCORP is based on a combinatorial optimization; and finally the network presented by Gallo et al. [27] (called GRNCORP2) that is a performance improvement of GRNCORP.

For this study, all the information stored in KEGG has been brought together in a single complex network. This global network (KEGG global network, KGN) is generated according to the knowledge gathered in each gene association network generated from* Saccharomyces cerevisiae* pathways. The aim of KGN is to conduct a global evaluation of the different networks to decide whether the networks contain biological knowledge or not. Specifically, the evaluation has been performed with level 2, according to the results obtained in the ROC analysis section. To compare the gene networks, only the relationships between genes contained in the input network and KGN have been considered. It is not possible to establish the quality of those interactions, because KEGG contains no information to ascertain whether the gene-gene interactions are biologically relevant or not.

In Table 2, the KGN rows, the global evaluation results, are shown. It is worth mentioning that two of the four networks obtain better validity results with the KGN because of the inclusion of a greater number of the indirect relationships (Hit_2_).

Once the results of the global KGN network are obtained, a specific functional biological analysis was performed. This analysis reveals whether the cell cycle's network describes a specific biological process or if the information is dispersed among all the pathways stored in the organism.

Therefore, all input networks have been compared with each of the 105 GNs obtained from* Saccharomyces cerevisiae* pathways. The most representative results were obtained with the* sce*04111 and* sce*04113 pathways, which represent the cell cycle and meiosis processes, respectively. These results are presented in the sce04111 and sce04113 rows of Table 2. They show that the networks store the majority of its biological information regarding the cell cycle metabolic pathway. For that reason, we obtain practically the same values using the cell cycle pathway or KGN for the evaluation.

Moreover, all the hits (Hits_1_ and Hits_2_) obtained in comparison to KGN are also found in cell cycle pathway sce04111. This information is summarized in Table 2 where the results of Hits_1_ and Hits_2_ are presented for all networks studied. As expected, the functionality found in the global evaluation is completed in relation to the yeast cell cycle process.

The input networks also contain biological information for the meiosis pathway (sce04113) (last row of Table 2). This does not contradict the previous result, since meiosis is related to the cell cycle [45], sharing gene-gene relationships.

The presented results show that our approach is able to identify the biological functionality that best describes the input network. Moreover, it is also able to find more related subprocesses.

## 4. Conclusions

A new methodology to score the biological validity of gene networks is proposed. Our presented approach, called GeneNetVal, entails identifying biological knowledge included in the input network. It is based on the gene-gene interaction relevance between the genes involved in the KEGG metabolic pathways. The method provides a complete and exhaustive conversion from a pathway to a gene association network. This approach also uses the concept of weak relationships between genes to present a new matching distance with different distance levels.

Three different experiments have been carried out. Firstly, our approach was compared to the classical use of KEGG to score the gene network validity. Comparisons were made for three different* Saccharomyces cerevisiae* complex networks. To demonstrate the robustness of the methodology, ROC analysis was performed for pure random and scale-free topologies. The results show that the proposal represents a significant improvement over the classical use of KEGG for assessing gene networks. Furthermore, it is possible to obtain better results by increasing the level of GeneNetVal in the comparison, where level 2 presents the best performance for all the experiments.

Secondly, a randomness study was performed. This study shows GeneNetVal is able to detect the loss of information in the input networks as the noise increases in them. Hence, our proposal distinguishes biologically correct from less correct networks.

Finally, the ability of GeneNetVal in finding a specific biological functionality was tested using some yeast cell cycle networks. The results show that our proposal is able to identify the main biological process described in an input network and other related processes.

## Figures and Tables

**Algorithm 1:**
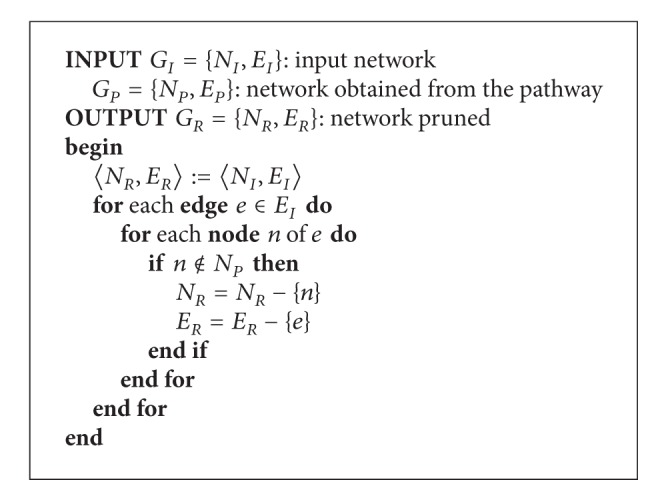
Pruning process.

**Table 1:** Possible interactions presented in KEGG are classified into two groups, gene relationships and others relationships. First the four types of gene relationships are presented.

KEGG relationships	Description
Gene relationships	
ECrel	Enzyme-enzyme relation
PPrel	Protein-protein interaction
GErel	Gene expression interaction
PCrel	Protein-compound interaction
Others	
compound-compound	Compound-compound interaction

**Table 2:** Most representative *Level*_2_ results found using our method assessing some yeast cell cycle networks. The results from GeneNetVal and the Hits levels 1 and 2 obtained from the evaluation are shown. Note that all similarities found with the KGN are also found for the cell cycle pathways network as expected.

	GeneNetVal_2_	Hit_1_	Hit_2_	GeneNetVal_2_	Hit_1_	Hit_2_
	Nariai et al. [26]			Gallo et al. [27]		
KGN	**0.852**	**35**	5	**0.65**	**4**	5
*sce*04111	**0.852**	**35**	5	**0.65**	**4**	5
*sce*04113	0.81	22	3	0.4	2	2

	Bulashevska and Eils [28]			Ponzoni et al. [29]		
KGN	**0.542**	**4**	5	**0.647**	**6**	10
*sce*04111	**0.458**	**4**	3	**0.618**	**6**	9
*sce*04113	0.2	0	2	0.417	1	1
